# Supplementary material for: Effect of Erythropoietin on the Expression of Murine Transferrin Receptor 2
Source: Int J Mol Sci. 2021 Jul 30;22(15):8209. doi: 10.3390/ijms22158209 (PMC8348427; doi:10.3390/ijms22158209)
Supplement: Supplementary file 1 [file ijms-22-08209-s001.zip › Figure S1.pdf]

Figure S1. Single injection of EPO upregulates *Epor* and *Cdc42bpa* mRNA in the spleen.

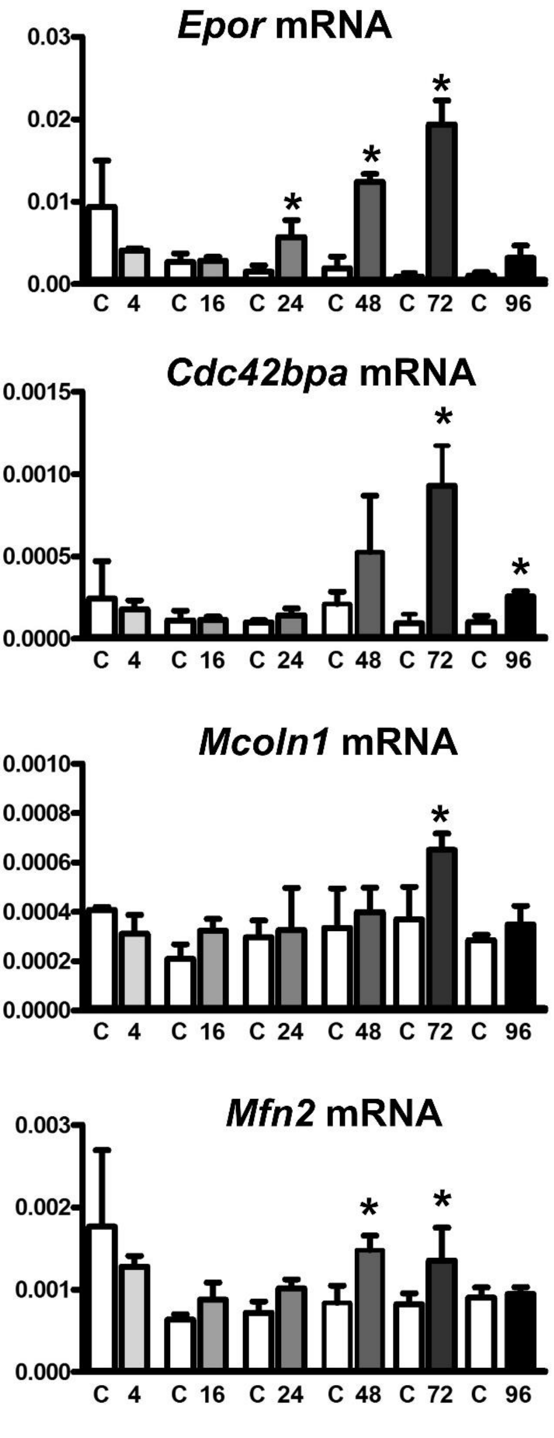

EPO was administered at 200 U/mouse and target gene expression was determined at the indicated time points (4, 16, 24, 48, 72 and 96 h post EPO injection) in groups of three control mice (C) and three EPO-treated mice. Target mRNA content is expressed relative to *Actb* expression, asterisks denote statistical significance between EPO-treated groups and the respective control groups.
