# Supplementary material for: Effect of Erythropoietin on the Expression of Murine Transferrin Receptor 2
Source: Int J Mol Sci. 2021 Jul 30;22(15):8209. doi: 10.3390/ijms22158209 (PMC8348427; doi:10.3390/ijms22158209)
Supplement: Supplementary file 1 [file ijms-22-08209-s001.zip › Figure S2.pdf]

Figure S2. Abcam ab80194 anti-TFR2 antibody does not detect TFR2 in serum.

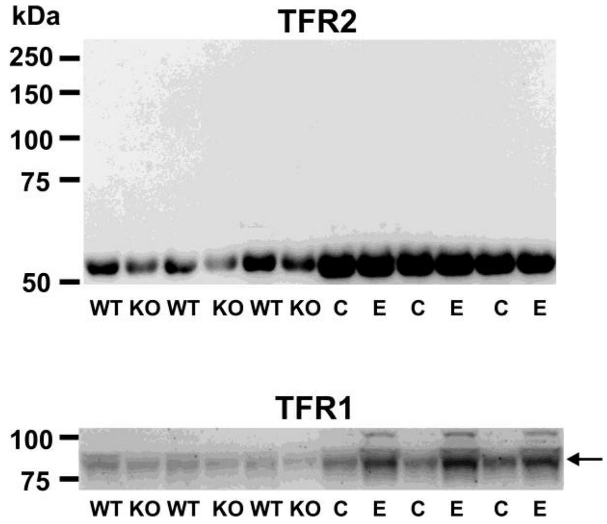

Serum from *Tfr2*+/+ mice (WT) and *Tfr2*-/- (KO) mice was depleted of albumin using the ProteoPrep Immunoaffinity Albumin and IgG depletion kit from Sigma. 40 µg of protein was separated on 8% acrylamide gel under reducing conditions, blotted and probed by Abcam ab80194 anti-TFR2 antibody (1:1000) and ab214039 anti-TFR1 antibody. No signal was detected in the expected region of soluble TFR2 size (approximately 90 kDa); the nature of the bands around 50 kDa is unclear. Serum from control C57BL/6 mice (C) and mice treated with EPO (E) 72 h prior to sacrifice was run on the same gel. Arrow denotes the plasma TFR1 band.
