# Supplementary material for: Effect of Erythropoietin on the Expression of Murine Transferrin Receptor 2
Source: Int J Mol Sci. 2021 Jul 30;22(15):8209. doi: 10.3390/ijms22158209 (PMC8348427; doi:10.3390/ijms22158209)
Supplement: Supplementary file 1 [file ijms-22-08209-s001.zip › Figure S3.pdf]

**Figure S4: Validation of the anti-TFR2 ab80194 antibody**

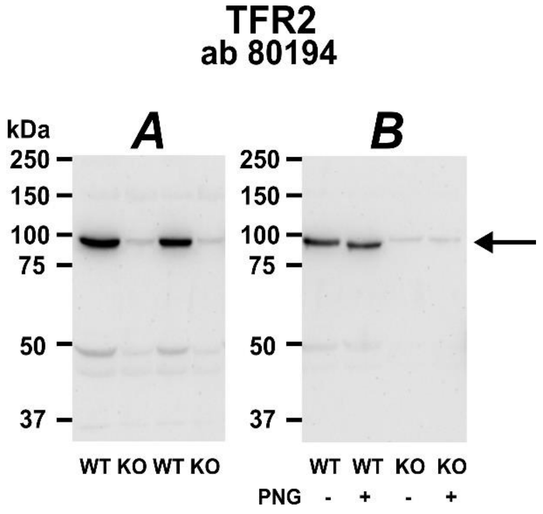


A: Detection of TFR2 in liver membranes from wild-type (WT) and *Tfr2-/-* (KO) mice. Arrow denotes the TFR2-specific band.

B: Effect of PNGase F treatment on samples shown in panel A. The TFR2-specific band shifts by approximately 4 kDa in treated samples (PNG +), which is in agreement with previously published data on TFR2 (Forejtnikova et al. 2010). The weak band seen in *Tfr2-/-* mice does not change following PNGase F treatment, indicating that it does not represent TFR2 or TFRC protein.
