# Supplementary material for: Effect of Erythropoietin on the Expression of Murine Transferrin Receptor 2
Source: Int J Mol Sci. 2021 Jul 30;22(15):8209. doi: 10.3390/ijms22158209 (PMC8348427; doi:10.3390/ijms22158209)
Supplement: Supplementary file 1 [file ijms-22-08209-s001.zip › Figure S4.pdf]

Figure S5. Gating strategy for FACS analysis.

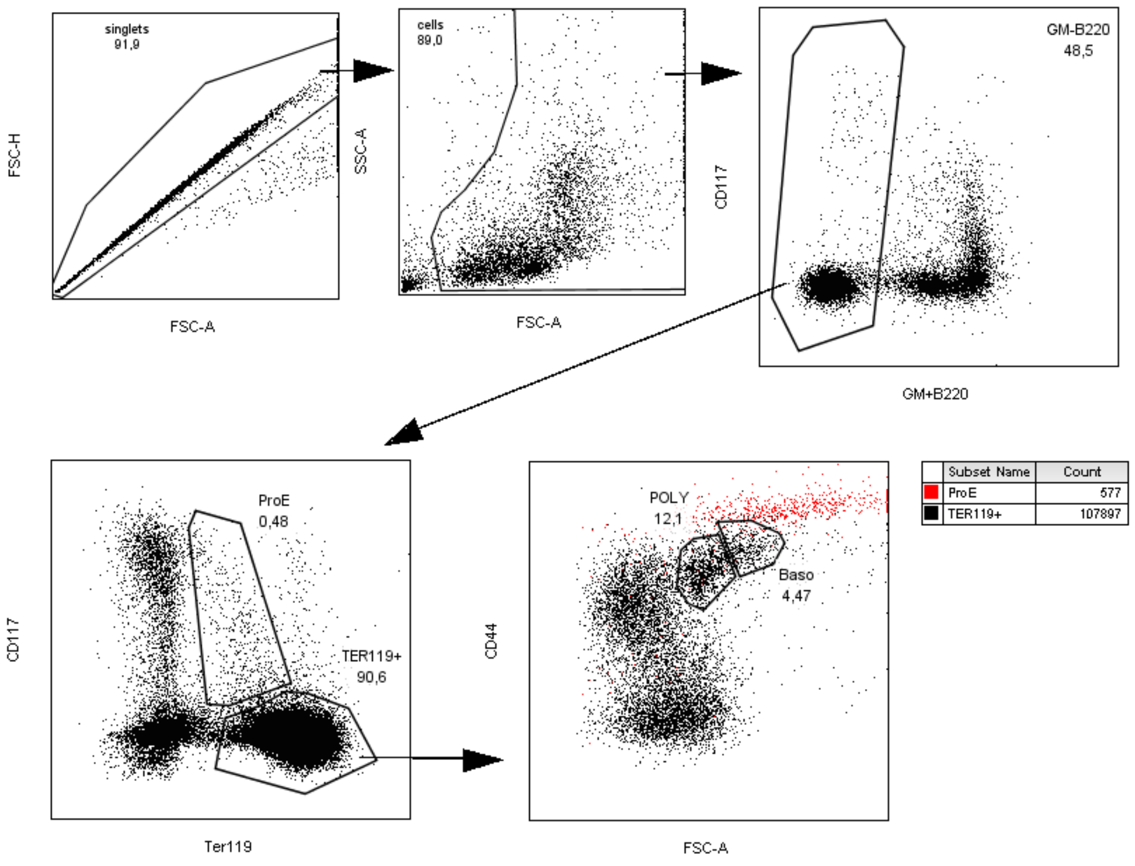

Sorting of erythrocyte precursors. Bone marrow and spleen cell populations were defined by immunophenotyping and forward (FSC) and side (SSC) scatter characteristics. Cells were sorted with a FACSAria IIu cell sorter (BD Biosciences) equipped with 489 nm (50 mW), 561 nm (100 mW), 638 nm (140 mW), 404 nm (100 Mw), and 355 nm (20 mW) lasers. Bone marrow and spleen cells were incubated with the indicated antibodies and sorted by the method of Chen et al [28]. Cells were isolated as described in Materials and Methods and sorted with the use of 70-micron integrated nozzle (with corresponding sheath pressure), under a “0-18-0” precision mode setup (yield mask 0, purity mask 18, phase mask 0). A compensation matrix was created by running single-stained control samples (automatic compensation). The compensation matrix was then checked and manually adjusted (if necessary) before each sorting procedure. Before cell sorting, “Checking cytometer performance” (CS&T) and “Determining the drop delay” (BD Biosciences Accudrop beads) procedures were executed. Sterile 1x PBS was used as sheath fluid. BD FACS Diva software version 6.1.2 was used for data acquisition.
