# Supplementary material for: Effect of Erythropoietin on the Expression of Murine Transferrin Receptor 2
Source: Int J Mol Sci. 2021 Jul 30;22(15):8209. doi: 10.3390/ijms22158209 (PMC8348427; doi:10.3390/ijms22158209)
Supplement: Supplementary file 1 [file ijms-22-08209-s001.zip › Table S1.pdf]

Table S1. Red blood cell parameters, spleen size and iron concentrations.

	0h	4h	16h	24h	48h	72h	96h
RBC	10.4±0.5	10.0±0.5	10.6±0.6	10.5±0.6	10.6±0.4	10.6±0.2	10.8±0.3
Hb	159±8	167±5	162±2	163±7	166±7	169±4	172±4*
HCT	46±2	45±5	45±3	46±2	49±1*	51+2*	51±1*
MCV	45±1	46±2	46±2	45±1	48±1*	47±1*	47±1*
Spleen	64± 6	83±22	78±11	77±9	127±26*	137±11*	111±10*
Fe (Pl)	20±4	21±3	18±6	33±7*	23±9	17±7	26±15

EPO was administered at 200 U/mouse; mice were sacrificed at indicated time intervals. RBC: Red blood cells (millions/µl); Hb: Hemoglobin (g/litre); HCT: Hematocrit (%); MCV: Mean cell volume (fl); Spleen: Spleen weight (mg); Fe (Pl): Plasma iron (µmol/l). Asterisk denotes statistical signifikance ($p<0.05$); n=4.
