# Supplementary material for: Effect of Erythropoietin on the Expression of Murine Transferrin Receptor 2
Source: Int J Mol Sci. 2021 Jul 30;22(15):8209. doi: 10.3390/ijms22158209 (PMC8348427; doi:10.3390/ijms22158209)
Supplement: Supplementary file 1 [file ijms-22-08209-s001.zip › Table S2.pdf]

Table S2

Primers used for PCR analysis

Actb:	F: GCTACAGCTTCACCACCACA	R: GGTCTTTACGGATGTCAACG
Cdc42bpa:	F: GGATGAACTTGTGGTTCCTGA	R: ACCCATGTGTGCTATGTGGTT
Epor:	F: GGACCCTGTGACTATGGATGA	R: CCCCGAACTGTAATCTGTTGA
Fam132b:	F: ATGCTGTTCGTCAAGCAGAGT	R: CCTTCAGCAGAACCTCAGATG
Hamp:	F: CTGAGCAGCACCACCTATCTC	R: TGGCTCTAGGCTATGTTTTGC
Id1:	F: CGAGGTGGTACTTGGTCTGTC	R: CTGCAGGTCCCTGATGTAGTC
Mcoln1:	F: ATGAGCCCATGTGACAAGTTC	R: AGGTGTCATCAGACCCATCAG
Mfn2:	F: ACTGACCTGGACCACCAAAG	R: TGGCTCTGCTCTGAAGTGAA
Tfr2:	F: CCCTATCTGGTCCTGATCACC	R: CAAGGAACCGGAGAAACATGG
Tfr1:	F: AAACTGGCTGCAGATGAAGAA	R: ATAGCCCAGGTAGCCACTCAT
