# Supplementary material for: Effect of Erythropoietin on the Expression of Murine Transferrin Receptor 2
Source: Int J Mol Sci. 2021 Jul 30;22(15):8209. doi: 10.3390/ijms22158209 (PMC8348427; doi:10.3390/ijms22158209)
Supplement: Supplementary file 1 [file ijms-22-08209-s001.zip › Table S3.pdf]

**Table S3**

**Reagents used in immunophenotyping of hematopoietic cells**

| Antibody | Clone | Producer | Fluorochrome |
|---|---|---|---|
| CD117 (c-kit) | 2b8 | http://www.biolegend.com/ | Brilliant Violet 421™ |
| CD71 | RI7217 | http://www.biolegend.com/ | FITC |
| CD44 | IM7 | http://www.biolegend.com/ | PE |
| CD45R/B220 | RA3-6B2 | http://www.biolegend.com/ | APC/Cy7 |
| Ly-6G(Gr-1) | RB6-8C5 | http://www.biolegend.com/ | APC/Cy7 |
| CD11b | M1/70 | http://www.biolegend.com/ | APC/Cy7 |
| TER-119/Erythroid Cells | TER119 | http://www.biolegend.com/ | Brilliant Violet 785™ |
